# Associations of Daily Walking Time With Pneumonia Mortality Among Elderly Individuals With or Without a Medical History of Myocardial Infarction or Stroke: Findings From the Japan Collaborative Cohort Study

**DOI:** 10.2188/jea.JE20170341

**Published:** 2019-06-05

**Authors:** Shigekazu Ukawa, Wenjing Zhao, Hiroshi Yatsuya, Kazumasa Yamagishi, Naohito Tanabe, Hiroyasu Iso, Akiko Tamakoshi

**Affiliations:** 1 Department of Public Health, Faculty of Medicine and Graduate School of Medicine, Hokkaido University, Hokkaido, Japan; 2 Research Unit of Advanced Interdisciplinary Care Science, Graduate School of Human Life Science, Osaka City University, Osaka, Japan; 3 Department of Public Health, Fujita Health University School of Medicine, Aichi, Japan; 4 Department of Public Health Medicine, Faculty of Medicine, University of Tsukuba, Tsukuba, Japan; 5 Department of Health and Nutrition, Faculty of Human Life Studies, University of Niigata Prefecture, Niigata, Japan; 6 Public Health, Department of Social and Environmental Medicine, Osaka University Graduate School of Medicine, Osaka University, Osaka, Japan

**Keywords:** walking, pneumonia, influenza, motor activity, epidemiology

## Abstract

**Background:**

The association between daily walking and pneumonia mortality, stratified by the presence of disease conditions, such as myocardial infarction (MI) or stroke, was investigated.

**Methods:**

The study participants were 22,280 Japanese individuals (9,067 men and 13,213 women) aged 65–79 years. Inverse propensity weighted competing risk model was used to calculate the hazard ratio (HR) and 95% confidence interval (CI) for pneumonia mortality.

**Results:**

After a median of 11.9 years of follow-up, 1,203 participants died of pneumonia. Participants who did not have a history of MI or stroke and who walked for 1 hour/day or more were less likely to die from pneumonia (HR 0.90; 95% CI, 0.82–0.98) than those walked for 0.5 hours/day. A similar inverse association of pneumonia and walking (0.5 hours/day) was observed among participants with a history of MI (HR 0.66; 95% CI, 0.48–0.90). Among the participants with a history of stroke, those who walked for 0.6–0.9 hours/day were less likely to die because of pneumonia (HR 0.65; 95% CI, 0.43–0.98).

**Conclusions:**

Regular walking for ≥1 hour/day may reduce the risk of pneumonia mortality in elderly individuals with or without cardiovascular disease history.

## BACKGROUND

Pneumonia is one of the leading causes of death in developed countries ranking sixth and eight in England^1^ and North America,^2^ respectively, among all underlying causes of death. Similarly, the combination of pneumonia and influenza ranks third among the leading causes of death in the Japanese elderly population, accounting for more than 561 deaths per 100,000 population annually.^3^ Indeed, several systematic reviews suggested that age ≥65 years is a risk factor of pneumonia.^4^^–^^6^ Previous cohort studies showed that walking^7^ or high-intensity physical activities^8^^,^^9^ were associated with a decreased risk of pneumonia. However, the elderly population often has underlying chronic diseases, such as myocardial infarction (MI) or stroke, which may prevent them from walking and increase the risk of pneumonia,^10^^,^^11^ and the association of walking with pneumonia may result from a combination of underlying conditions. Therefore, this study aimed to investigate whether daily walking time was associated with pneumonia mortality in Japanese participants aged 65–79 years with or without a medical history of MI or stroke.

## METHODS

### Study population and data collection

The Japan Collaborative Cohort Study for Evaluation of Cancer Risk (JACC Study), which was established in 1988–1990, has been described in detail elsewhere.^12^^,^^13^ Briefly, 110,585 inhabitants (46,395 men and 64,190 women) aged 40–79 years from 45 areas in Japan were enrolled into the study. In the present study, the overall number of baseline participants (aged 65–79 years) was 29,956 (12,196 men and 17,760 women). Data were collected through a self-administered questionnaire, and a response rate of 83% was observed. Information on the average number of daily walking hours and other lifestyle factors was obtained from the baseline questionnaire. Participants were asked about the average daily time spent walking: “How long on average do you spend walking indoors or outside on a daily basis?”, and the possible responses were: “<0.5 hours/day”, “0.5 hours/day,” “0.6–0.9 hours/day,” and “≥1.0 hours/day.”

Of the original cohort members, 5,492 participants in six areas were excluded because the questionnaire that was used did not include data on the average number of daily walking hours. Furthermore, 2,184 participants from other areas with missing data on the average daily walking hours were excluded. Consequently, 22,280 participants (9,067 men and 13,213 women) were included in the present study.

### Follow-up

The date and cause of death were confirmed via death certificates and coded according to the International Classification of Diseases, 10^th^ Revision (ICD-10). The primary outcome of the present study was death due to pneumonia or influenza (J9–18, J69). Participants who moved away from the study area during the study period were treated as censored cases.

### Statistical analysis

We used four statistical models to calculate multivariate-adjusted hazard ratios (HRs) and confidence intervals (CIs) for pneumonia-related mortality.^14^^,^^15^ First, we conducted multivariate adjustment using an inverse probability weighting (IPW) method based on generalized propensity scores because of a relatively small number of pneumonia deaths.^16^ This approach is a statistical alternative to propensity score matching that balances the confounders in non-randomized studies. To develop the generalized propensity score, we conducted a multinomial logistic regression analysis for the four walking categories using all the demographic information,^17^ including, age (as a continuous variable), sex (male or female), smoking status (never, former, current smoking, or unknown), alcohol drinking status (never, former, current alcohol drinker, or unknown), body mass index (BMI; <18.5, 18.5–24.9, ≥25.0 kg/m^2^, or unknown), educational level (school age up to age 15 years, 15–18 years, ≥19 years, or unknown), marital status (single, married, divorced/widowed, or unknown), depressive tendency (defined later; presence, absence, or unknown), sleep duration (<6.5 hours/day, 6.5–8.4 hours/day, ≥8.5 hours/day, or unknown), and a history of cancer (yes or no/unknown), diabetes mellitus (yes or no/unknown), kidney diseases (yes or no/unknown), and asthma (yes or no/unknown). Four psychological or behavioral items from the baseline questionnaire were used to quantify depressive tendency.^18^ These items are the following: “Do you think your life is meaningful?”, “Do you think you make decisions quickly?”, “Are you enjoying your life?”, and “Do you feel others rely very much on you?”. Participants with two or more psychological or behavioral items were considered to have a depressive tendency. Second, to assess for covariate balance, we showed the propensity score overlap through kernel density plots. Third, we then used an IPW Cox proportional hazards model.^19^ Finally, we utilized a competing risk model,^20^ in which death caused by factors other than pneumonia was considered as a competing risk. Linear trends in mortality risks were assessed based on four categories of walking hours per day, which were used as numeric variables. To avoid an inverse causal relationship, we considered the participants who walked for 0.5 hours/day as ambulatory,^21^ and they were included in the reference group. All statistical analyses were conducted separately among participants with or without a history of MI or stroke. An alpha level of 0.05 was considered statistically significant. All statistical analyses were performed using SAS 9.4 (SAS Institute Inc., Cary, NC, USA). The graphs were drawn using JMP 12.0.2 (SAS Institute Inc.).

## RESULTS

Of the 22,280 participants, 1,210, 604, and 80 participants had a history of MI, stroke, and both MI and stroke, respectively. The baseline mean age of the participants was 70.5 (standard deviation [SD], 4.1) years (men, 70.6 [SD, 4.1] years; women, 70.4 [SD, 4.1] years).

The baseline characteristics of the participants with or without a history of MI or stroke according to daily walking time are shown in Table 1. About 50.4% of participants without a history of MI and stroke, 41.8% of participants with a history of MI, and 33.9% of the participants with a history of stroke walked >1 hour daily. Among the participants with or without a history of MI or stroke, those who walked >1 hour/day were younger in age, women, and current drinkers; these participants also had a BMI of 18.5–25.0 kg/m^2^ and sleep duration of 6.5–8.4 hours/day. Moreover, they were less likely to study in college or obtain a diploma and have a depressive tendency and history of diabetes mellitus and cancer compared with those who walked for 0.5 hours/day. The differences of all the potential confounders among the four categories of walking were reduced after weighting (eFigure 1).

**Table 1.** Baseline characteristics of participants with/without history of MI or stroke according to daily walking time (hours/day)

Characteristics	Category	**Without history of MI and stroke**				**With history of MI**				**With history of stroke**			
		
		<0.5 (*n* = 1,983)	0.5 (*n* = 3,753)	0.6–0.9 (*n* = 4,426)	≥1 (*n* = 10,314)	<0.5 (*n* = 145)	0.5 (*n* = 303)	0.6–0.9 (*n* = 303)	≥1 (*n* = 539)	<0.5 (*n* = 146)	0.5 (*n* = 166)	0.6–0.9 (*n* = 140)	≥1 (*n* = 232)
Age, years		70.7 ± 4.3	70.7 ± 4.2	70.5 ± 4.1	70.2 ± 4.0	71.2 ± 4.1	71.4 ± 4.1	71.4 ± 4.3	70.8 ± 4.3	71.8 ± 4.2	71.8 ± 4.3	71.1 ± 4.1	71.0 ± 3.5
Sex	Male	43.2	39.8	39.5	39.8	45.5	41.6	42.9	41.2	62.3	54.2	60.7	61.6
Body mass index, kg/m^2^	<18.5	11.6	10.8	9.6	9.6	9.0	9.2	8.9	8.1	12.3	14.5	8.6	9.5
	18.5–24.9	59.2	61.5	65.2	66.1	58.6	65.4	64.0	66.6	56.9	59.0	67.1	66.0
	≥25.0	19.0	18.0	16.5	15.6	17.9	18.2	16.2	16.7	18.5	13.9	18.6	13.8
College education	Yes	0.4	0.4	0.6	0.3	0.7	1.0	0.7	0.2	2.1	0.6	0	0
Current smoker	Yes	20.7	19.2	18.1	19.8	24.1	17.8	14.9	16.0	19.2	15.7	14.3	19.4
Current alcohol drinker	Yes	30.9	34.4	34.7	35.0	26.9	39.3	37.3	35.1	23.3	29.5	33.6	44.0
Sleep duration, hours/day	6.5–8.4	54.6	56.8	59.7	59.7	38.6	55.5	53.5	54.0	39.7	48.2	55.0	52.6
Depressive tendency	Yes	12.8	7.1	6.4	5.6	15.9	8.3	7.6	6.7	33.6	19.3	10.0	12.5
History of													
Diabetes mellitus	Yes	7.3	8.5	7.0	5.4	15.2	16.2	12.5	9.1	14.4	14.5	7.9	11.2
Kidney disease	Yes	3.8	4.7	4.9	4.3	10.3	11.2	8.6	8.7	6.2	10.2	7.9	6.5
Asthma	Yes	1.8	1.3	0.9	0.8	0.7	1.3	1.9	0	2.1	0	0	0.9
Cancer	Yes	2.8	2.5	2.7	1.8	4.8	5.9	4.3	3.5	3.4	7.2	2.1	4.7

MI, myocardial infarction.

Values are expressed as mean ± standard deviation or percentage.

The median follow-up period was 11.9 years. For 306,578 person-years of follow-up (109,078 person-years for men and 197,500 person-years for women), 1,203 participants (731 men and 472 women) died from pneumonia, 1,367 left the study area, and 9,753 died because of factors other than pneumonia. The HRs of pneumonia mortality according to walking time are shown in Table 2. Participants without a history of MI or stroke who walked for more than 1 hour/day were less likely to die because of pneumonia (HR 0.90; 95% CI, 0.82–0.98; *P* for trend < 0.001) compared with those who walked for 0.5 hours/day. Similarly, decreased HRs were observed among participants with a history of MI (HR 0.66; 95% CI, 0.48–0.90; *P* for trend = 0.02). Among the participants with a history of stroke, those who walked for more than 0.6–0.9 hours/day were less likely to die because of pneumonia compared with those who walked for 0.5 hour/day (HR 0.65; 95% CI, 0.43–0.98). In contrast, the death of individuals who walked ≥1 hour/day was not associated with pneumonia (HR 1.15; 95% CI, 0.81–1.63).

**Table 2.** Hazard ratios of pneumonia mortality according to walking time among participants with/without a history of MI or stroke

Walking time, hours/day	PY	*n*	HR (95% CI)^a^	HR (95% CI)^b^
**Without history of MI and stroke**				
<0.5	22,288	141	1.44 (1.15, 1.81)^*^	1.33 (1.22, 1.43)^*^
0.5	46,800	208	Ref	Ref
0.6–0.9	57,351	230	0.95 (0.78, 1.14)	0.97 (0.89, 1.05)
≥1	142,023	489	0.84 (0.71, 0.99)^*^	0.90 (0.82, 0.98)^*^
*P* for linear trend			<0.001	<0.001
**With history of MI**				
<0.5	1,340	12	0.88 (0.40, 1.96)	0.93 (0.70, 1.23)
0.5	2,850	21	Ref	Ref
0.6–0.9	3,030	20	0.81 (0.44, 1.50)	0.90 (0.68, 1.21)
≥1	5,991	27	0.55 (0.31, 0.99)^*^	0.66 (0.48, 0.90)^*^
*P* for linear trend			0.18	0.02
**With history of stroke**				
<0.5	1,011	21	2.05 (1.01, 4.20)^*^	1.66 (1.19, 3.33)^*^
0.5	1,537	14	Ref	Ref
0.6–0.9	1,456	8	0.57 (0.24, 1.38)	0.65 (0.43, 0.98)^*^
≥1	2,535	22	0.89 (0.43, 1.83)	1.15 (0.81, 1.63)
*P* for linear trend			0.006	0.008

CI, confidence interval; CVD, cardiovascular disease; HR, hazard ratio; MI, myocardial infarction; PY, person-year.

*P* for trend was calculated across the categories of walking time. The generalized propensity scores were calculated by a multinomial logistic regression analysis for the four walking categories using the demographic information, including, age, sex, smoking status, alcohol drinking status, body mass index, educational level, marital status, depressive tendency, sleep duration, and a history of cancer, diabetes mellitus, kidney diseases, and asthma.

^*^P < 0.05.

^a^Cox proportional hazard model with inverse propensity weighting.

^b^Competing risk model with inverse propensity weighting.

## DISCUSSION

In this large cohort study, participants without a history of MI or stroke who walked for ≥1 hour/day were less likely to die from pneumonia than those who walked for 0.5 hours/day. Similar results were obtained among participants with a history of MI. For participants with a history of stroke, those who walked for 0.6–0.9 hours/day were less likely to die from pneumonia than those who walked for 0.5 hours/day. However, this inverse association was not observed in participants with a history of stroke who walked ≥1 hour/day.

Participants with or without a history of MI or stroke who walk for a longer number of hours per day were less likely to die from pneumonia compared with those who walk for a shorter number of hours per day. Our finding shows a reduced incidence of pneumonia^8^ and pneumonia mortality^7^^,^^9^ in community residents with moderate physical activities, and this finding is similar to the results obtained from three previous studies. Whether elderly individuals with coronary heart disease are more susceptible to pneumonia than those without coronary heart disease is not clear.^22^ Two studies, including a cohort study^23^ and nested case-control study,^10^ suggested that elderly individuals with chronic cardiovascular disease were more likely to develop pneumonia compared with those without the disease condition (HR 1.46; 95% CI, 1.16–1.84 and HR 1.68; 95% CI, 1.58–1.77, respectively). These individuals may acquire pneumonia because of deteriorated immunological responses.^24^ Furthermore, since pneumonia itself causes cardiac problems,^25^ such as left ventricular dysfunction^26^^,^^27^ or increased cardiac arrhythmias,^28^^,^^29^ those who experienced cardiac complications along with pneumonia were more likely to die.^30^ Therefore, walking may be important for individuals with heart disease because it enhances mucosal immune function in elderly individuals.^31^ Furthermore, moderate physical activities, such as walking, enhance the immune function by increasing the activities of macrophages, natural killer cells,^32^ and neutrophils and regulating cytokines.^33^^,^^34^ However, caution must be taken into consideration. Although rare, some patients with coronary artery disease have sudden cardiac death during exercise, particularly habitually sedentary adults.^35^ Thus, elderly individuals with a history of coronary artery disease should obtain an exercise prescription from physicians to avoid inappropriate physical activities.

A population-based cohort study suggested that elderly participants with a history of stroke were 1.26 (95% CI, 1.08–1.48) times more likely to be diagnosed with pneumonia than those without stroke after a 3-year follow-up.^11^ In the present study, participants with a history of stroke who walked for 0.6–0.9 hours/day were less likely to die from pneumonia compared with those who walked for 0.5 hours/day. This benefit was not observed among those who walked ≥1 hour/day. However, the mechanisms explaining why a longer walking time had no additional benefits for elderly individuals with stroke was unclear. Although a meta-analysis revealed that aerobic exercise training during the chronic stage of stroke recovery has beneficial effects on cardiorespiratory health,^36^ the oxygen cost of walking among people with history of stroke is 2-fold higher than that reported for non-stroke participants.^37^ Furthermore, other biological changes that may negatively affect cardiorespiratory health after stroke include elevated systemic levels of proinflammatory markers, abnormal glucose levels and insulin metabolism, impaired autonomic control, and respiratory dysfunction.^38^ In the context of age- and disease-related heterogeneity in cardiorespiratory capacity and medical comorbidities, walking duration ≥1 hour/day might be too much for elderly individuals with a history of stroke. However, to clarify an appropriate duration and time of physical activities for elderly individuals with a history of stroke, further epidemiological studies should be conducted.

The strengths of the present study include its prospective cohort design, long follow-up period, and an inclusion of participants from Japan. Several limitations should be discussed. First, we obtained information on the daily walking time through self-report, and that information was not validated. Therefore, some misclassifications were possibly included in our results.^39^ The use of an accelerometer^40^ can provide more reliable results. Second, the effects of the residual confounding factors were not completely excluded. Third, information on the duration and severity of MI and stroke were not available. These conditions may affect walking ability, capacity, or habit. Fourth, we obtained information retrospectively via medical histories; therefore, some misclassification could be included in our results.

### Conclusion

This large-scale cohort study demonstrated that walking at least ≥1 hour/day reduced the risk of pneumonia mortality among elderly individuals with or without a medical history of MI. Our findings suggest that regular walking may be beneficial in reducing the risk of pneumonia mortality in the elderly population.

## APPENDIX A. SUPPLEMENTARY DATA

eFigure 1. Kernel smoothed density estimate of propensity for walking 0.5 hours/day among participant with/without a medical history of myocardial infarction or stroke. A, B: Participants without a history of myocardial infarction and stroke; C, D: Participants with a history of myocardial infarction; E, F: Participants with a history of stroke. Kernel smoothed density estimates show improved overlap of propensity for walking 0.5 hours/day after inverse probability weighting.

## APPENDIX B. MEMBERS OF JAPAN COLLABORATIVE COHORT STUDY GROUP

The present members of the JACC Study Group who coauthored this paper are: Dr. Akiko Tamakoshi (present chairperson of the study group), Hokkaido University Graduate School of Medicine; Drs. Mitsuru Mori and Fumio Sakauchi, Sapporo Medical University School of Medicine; Dr. Yutaka Motohashi, Akita University School of Medicine; Dr. Ichiro Tsuji, Tohoku University Graduate School of Medicine; Dr. Yosikazu Nakamura, Jichi Medical School; Dr. Hiroyasu Iso, Osaka University School of Medicine; Dr. Haruo Mikami, Chiba Cancer Center; Dr. Michiko Kurosawa, Juntendo University School of Medicine; Dr. Yoshiharu Hoshiyama, Yokohama Soei University; Dr. Naohito Tanabe, University of Niigata Prefecture; Dr. Koji Tamakoshi, Nagoya University Graduate School of Health Science; Dr. Kenji Wakai, Nagoya University Graduate School of Medicine; Dr. Shinkan Tokudome, National Institute of Health and Nutrition; Dr. Koji Suzuki, Fujita Health University School of Health Sciences; Dr. Shuji Hashimoto, Fujita Health University School of Medicine; Dr. Shogo Kikuchi, Aichi Medical University School of Medicine; Dr. Yasuhiko Wada, Faculty of Nutrition, University of Kochi; Dr. Takashi Kawamura, Kyoto University Center for Student Health; Dr. Yoshiyuki Watanabe, Kyoto Prefectural University of Medicine Graduate School of Medical Science; Dr. Kotaro Ozasa, Radiation Effects Research Foundation; Dr. Tsuneharu Miki, Kyoto Prefectural University of Medicine Graduate School of Medical Science; Dr. Chigusa Date, School of Human Science and Environment, University of Hyogo; Dr. Kiyomi Sakata, Iwate Medical University; Dr. Yoichi Kurozawa, Tottori University Faculty of Medicine; Drs. Takesumi Yoshimura and Yoshihisa Fujino, University of Occupational and Environmental Health; Dr. Akira Shibata, Kurume University; Dr. Naoyuki Okamoto, Kanagawa Cancer Center; and Dr. Hideo Shio, Moriyama Municipal Hospital.
