# Supplementary material for: Associations of Daily Walking Time With Pneumonia Mortality Among Elderly Individuals With or Without a Medical History of Myocardial Infarction or Stroke: Findings From the Japan Collaborative Cohort Study
Source: J Epidemiol. 2019 Jun 5;29(6):233–7. doi: 10.2188/jea.JE20170341 (PMC6522391; doi:10.2188/jea.JE20170341)
Supplement: Supplementary file 1 [file je-29-233-s001.pdf]

A. Unadjusted estimates

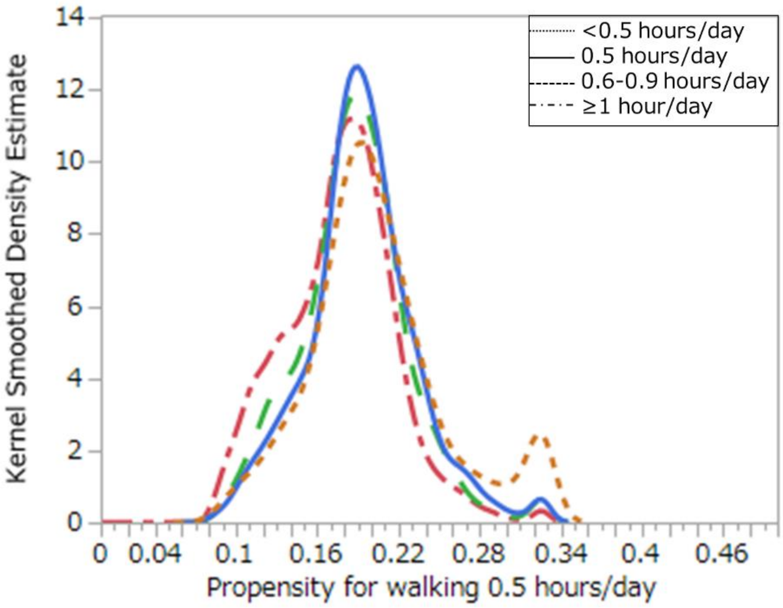

B. Adjusted estimates

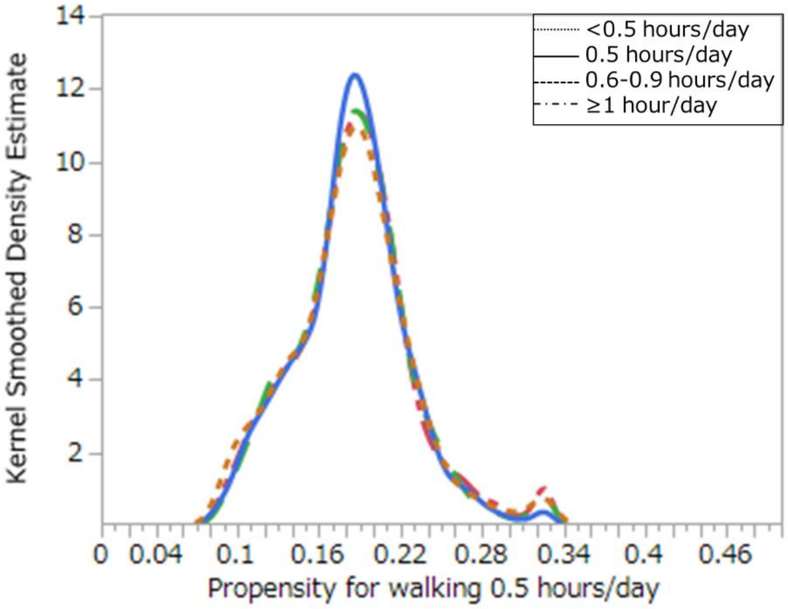

C. Unadjusted estimates

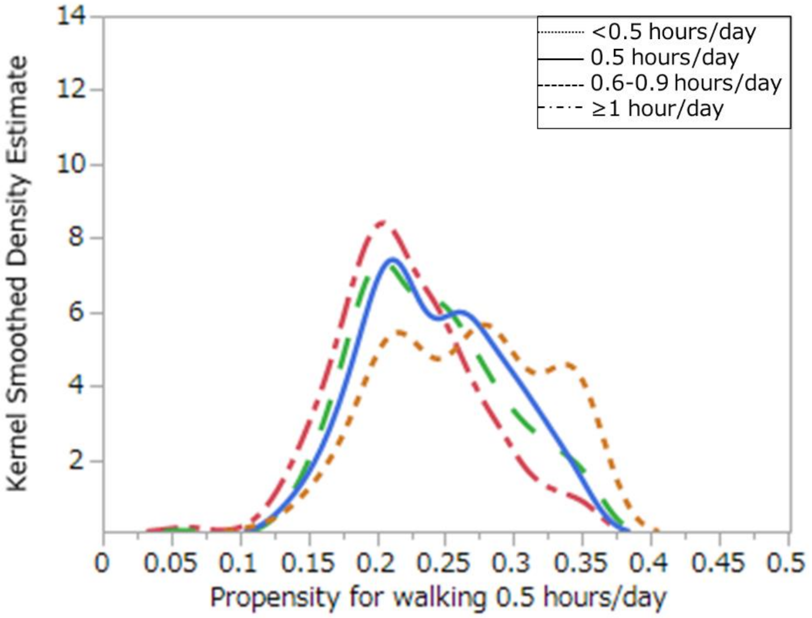

D. Adjusted estimates

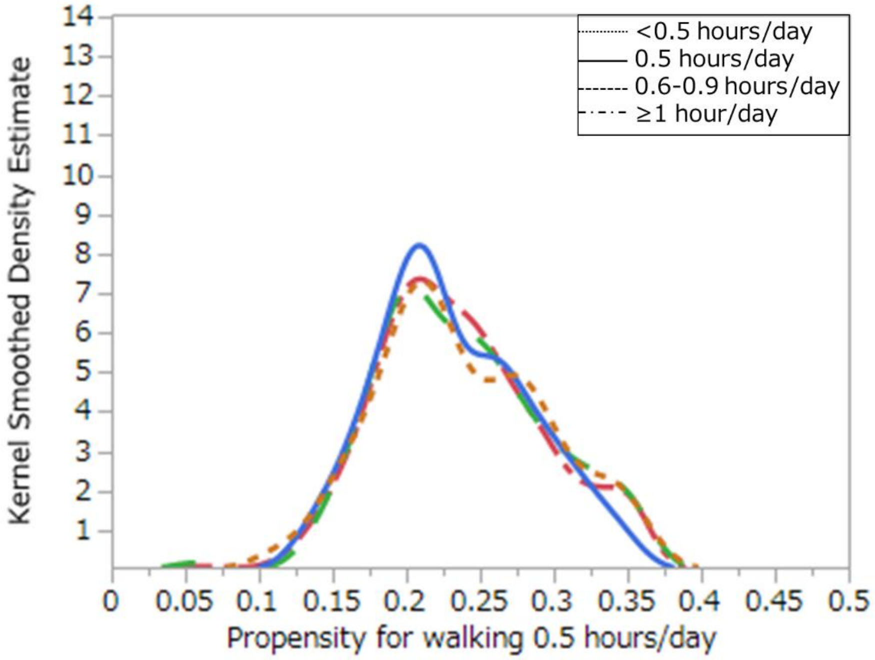

E. Unadjusted estimates

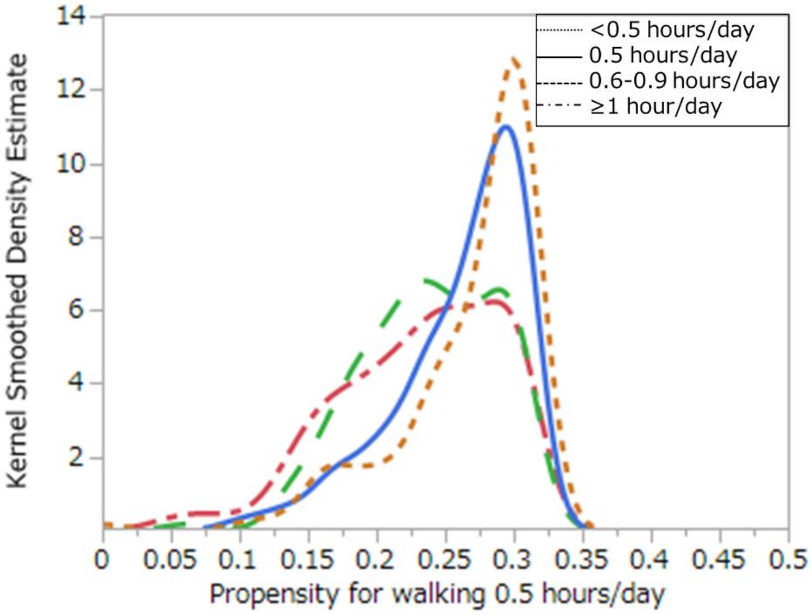

F. Adjusted estimates

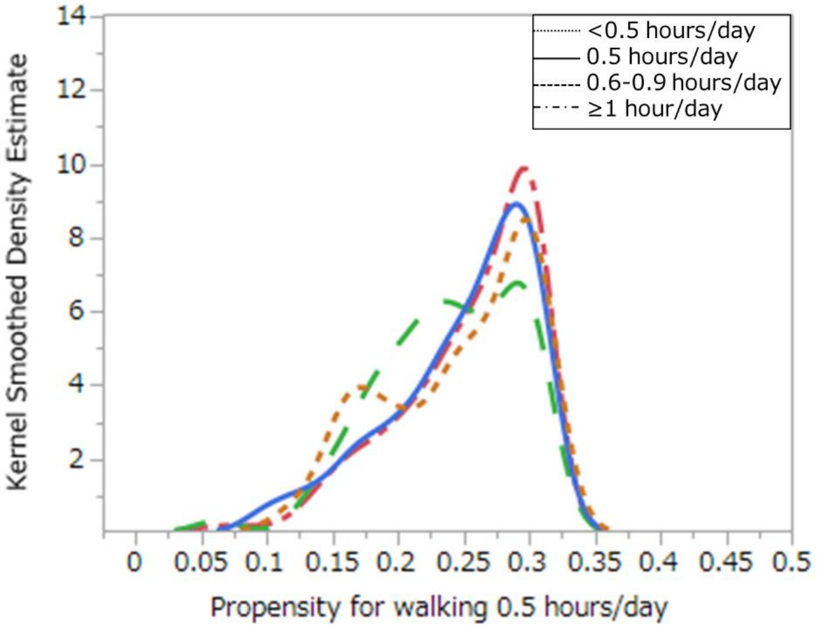

eFigure 1. Kernel smoothed density estimate of propensity for walking 0.5 hours/day among participant with/without a medical history of myocardial infarction or stroke. A, B: Participants without a history of myocardial infarction and stroke; C, D: Participants with a history of myocardial infarction; E, F: Participants with a history of stroke. Kernel smoothed density estimates show improved overlap of propensity for walking 0.5 hours/day after inverse probability weighting.
